# YBX3 Mediates the Metastasis of Nasopharyngeal Carcinoma *via* PI3K/AKT Signaling

**DOI:** 10.3389/fonc.2021.617621

**Published:** 2021-03-17

**Authors:** Xiaoqin Fan, Xina Xie, Ming Yang, Yujie Wang, Hanwei Wu, Tingting Deng, Xin Weng, Weiping Wen, Guohui Nie

**Affiliations:** ^1^ Department of Otolaryngology, The First Affiliated Hospital of Sun Yat-Sen University, Guangzhou, China; ^2^ Department of Otolaryngology, Shenzhen Second People’s Hospital, The First Affiliated Hospital of Shenzhen University, Shenzhen, China; ^3^ Guangdong Key Laboratory of Systems Biology and Synthetic Biology for Urogenital Tumors, Institute of Translational Medicine, Shenzhen Second People’s Hospital, First Affiliated Hospital of Shenzhen University, Shenzhen, China; ^4^ Department of Otolaryngology, Shenzhen First People’s Hospital, The Affiliated Hospital of Jinan University, Guangzhou, China; ^5^ Department of Pathology, Shenzhen Second People’s Hospital, The First Affiliated Hospital of Shenzhen University, Shenzhen, China

**Keywords:** nasopharyngeal carcinoma, Y-box protein 3, tumor metastasis, PI3K/AKT signaling pathway, MMP1, epithelial-to-mesenchymal transition (EMT)

## Abstract

The metastasis of nasopharyngeal carcinoma (NPC) is a complex process associated with oncogenic dysfunction, the deciphering of which remains a challenge and requires more in-depth studies. Y-box protein 3 (YBX3) is a DNA/RNA binding protein associated with gene transcription, DNA repair, and the progression of various diseases. However, whether and how YBX3 affects the metastasis of NPC remains unknown. Thus, in this study, we aimed to investigate the role of YBX3 in the metastasis of NPC and determine its underlying mechanism. Interestingly, it was found that the expression of YBX3, which was associated with NPC metastasis, was upregulated in the clinical NPC tissues and cell lines. Moreover, we found that knockdown of YBX3 expression by lentivirus shRNA significantly suppressed NPC cells migration *in vitro* and metastasis *in vivo.* Mechanistically, RNA sequencing results suggested that the genes regulated by YBX3 were significantly enriched in cell adhesion molecules, cAMP signaling pathway, calcium signaling pathway, focal adhesion, PI3K/AKT signaling pathway, Ras signaling pathway, Rap1 signaling pathway, NF-κB signaling pathway, and Chemokine signaling pathway. Of these, PI3K/AKT signaling pathway contained the most genes. Accordingly, YBX3 knockdown decreased the activation of PI3K/AKT signaling pathway, thereby inhibit epithelial-to-mesenchymal transition (EMT) and MMP1. These results have demonstrated that YBX3 are involved in the metastasis of NPC through regulating PI3K/AKT signaling pathway, and serve as a potential therapeutic target for patients with NPC.

## Introduction

Nasopharyngeal carcinoma (NPC) is a head and neck tumor whose incidence is higher in Southern China, Mediterranean Africa and the Middle East (1–3). Although the morbidity and mortality of NPC are lower than other malignant tumors, patients with recurrent and metastatic NPC still have poor outcomes and survival rates (4–6). Therefore, metastasis is the main cause of death in patients with NPC. Previous studies suggested that the metastasis of NPC is a complex process that involves a number of factors and cell signaling pathways (7, 8). However, the molecular mechanism underlying NPC metastasis remain elusive.

As one group of DNA/RNA binding proteins, Y-box protein family is characterized by the presence of the cold shock domains(CSDs), which is highly conserved from bacteria to humans (9). Y-box protein family have diverse functions, such as regulation of transcription, splicing, translation, and mRNA stability (10, 11). And Y-box protein family mainly contains three genes in humans, including Y-box protein 1 (YBX1), Y-box protein 2 (YBX2), and Y-box protein 3 (YBX3). YBX3 (also referred to as DBPA, ZONAB, or CSDA) a member of the Y-box protein family, is a 60 kDa protein first described by Sakura et al. and cloned by Kudo et al. (12, 13). YBX3 is ubiquitous expressed in human skeletal muscle, heart, and decidual cells, which is thought to a multifunctional epithelial-specific protein involved in gene transcription, DNA repair, and interaction with other proteins (14). Previous studies have demonstrated that YBX3 plays a role in gene transcription and translation of target genes that involved in cell survival, proliferation, and differentiation (15, 16). Recently, growing evidences have implicated that YBX3 also plays very important functions in the progress of various diseases, such as cancers (17, 18), nephropathy (19), and gastrointestinal diseases (20). For example, the YBX3 was upregulated in various tumor cells, and this upregulation is related to the growth of tumor cells and resistance to chemotherapy (21). Additionally, YBX3 was identified as a potential therapeutic target gene, which was significantly correlated with OS and metastasis in Hepatocellular carcinoma (22). Therefore, YBX3 was considered as a cancer prognosis marker. However, the gene expression, biological functions, and prognostic values of YBX3 in NPC metastasis are still not clearly understood.

In the present study, we found that YBX3 could promote NPC cellular motility *in vitro* and metastasis *in vivo*. In addition, YBX3 induced EMT by activating the PI3K/AKT pathway. Importantly, elevated the expression of YBX3 in NPC was closely associated with metastasis. These findings will provide a reliable molecular biomarker for the metastatic NPC.

## Materials and Methods

### Cell Lines and Reagents

The NP460 cell line (human normal nasopharyngeal epithelial cell) was kindly provided by Professor Tsao’s research group from Hong Kong University. The cells were cultured in 1:1 ratio of Defined Keratinocyte-SFM (DKSFM) medium and Epilife medium supplemented with growth factors EDGS (Gibico). The human nasopharyngeal carcinoma cell line 5-8F was cultured as previously described (23). The potentially high metastasis nasopharyngeal carcinoma cell line S18 was kindly provided by Professor Qian of the Cancer Center of Sun Yat-sen University. Cells were cultured in DMEM (Hyclone) supplemented with 10% Fetal Bovine Serum (FBS) (Gibico) and 1% Penicillin-Streptomycin solution (Hyclone). All cell lines were cultured at 37°C in a humidified incubator with 5% CO_2_.

Reagents were sourced commercially as follows: YBX3 antibody was purchased from Immuno-Biological Laboratory; GAPDH, AKT, phospho-AKT, E-cadherin, Vimentin, MMP1 antibodies were purchased from Protein Technology; PI3K, phosphor-PI3K were purchased from Cell Signaling Technology.

### Clinical Samples

All clinical samples were obtained under a protocol approved by the Ethics Committee of the Institutional Review Board of Shenzhen Second People’s Hospital. Informed consent was obtained for the use of patient-derived tissue samples for experimentation. Paraffin-embedded normal nasopharyngeal tissues (n = 6) and nasopharyngeal carcinoma tissues (n = 6) were obtained in the Department of Pathology of Shenzhen Second People’s Hospital. These cases were identified according to the World Health Organization Histological Typing (WHOHT). The paraffin-embedded tissue sections were examined by using hematoxylin and eosin (H&E) or YBX3 immunohistochemical staining. Fresh normal nasopharyngeal tissues (n = 6) and nasopharyngeal carcinoma tissues (n = 6) were obtained by nasal endoscopy in the Department of Otolaryngology of Shenzhen Second People’s Hospital.

### Computational Analysis and Database

The mRNA expression levels of YBX3 in NPC tissues and normal tissues were analyzed using ONCOMINE gene expression array database (www.oncomine.org) (24). The microarray data of NPC tumor tissues with and without distant metastasis were downloaded from the GEO database (Accession number: GSE103611) (25). The RNA Sequencing analysis data of 5-8F cells transfecting by lentivirus shRNA-YBX3 or shRNA-control were uploaded to the GEO database (Accession number: GSE160245).

### Lentiviral YBX3-shRNA Transfection and Cell Culture

The 5-8F and S18 cells were transfected by using lentivirus-packing control or YBX3-shRNAs technology (GenePharma). The cells were plated onto six-well plates and transfected with the YBX3-shRNAs or non-targeting control according to manufacturer’s instruction. The 2 ug/ml dose of puromycin (Solarbio) was used to select stable colonies. The transfection efficiency was evaluated using Q-PCR and WB analyses.

### Animal Experiments

All the animal experiments complied with the ARRIVE guidelines and experimental procedures were approved by the Animal Ethics Committee of Shenzhen University. Six-weeks-old female BALB/c nude mice were purchased from Beijing Huafukang Biotechnology and housed under pathogen-free conditions. For tumor metastasis models, 1 × 10^5^ cells in 20 µl of ice-cold PBS of YBX3 control or YBX3 shRNA 5-8F/S18 transfected cells were injected into the tail vein of each mouse. All mice were sacrificed on day 30, then the lung tissues were fixed in 4% paraformaldehyde (PFA) for histopathological analysis.

### Western Blot Analysis

The cells and tumor tissues were lysed using RIPA buffer (Beyotime) with protease and phosphatase inhibitors (Roche). Total protein concentration was estimated using the BCA quantitative assay (Protein Technology). Approximately 10 ug of protein samples was resolved using SDS-PAGE and transferred onto 0.2 μm PVDF membranes (Millipore). Membranes were blocked by 5% skim milk in TBST for 1 h and probed with primary antibodies against YBX3 (Immuno-Biological Laboratory); GAPDH, AKT, phospho-AKT, E-cadherin, Vimentin, and MMP1 (Protein Technology); PI3K and phosphor-PI3K (Cell Signaling Technology) at 1:1,000 dilution at 4°C overnight, washed with TBST three times, and then incubated with secondary antibodies against Anti-mouse IgG and Anti-rabbit IgG HRB-linked antibody (Cell Signaling Technology) at 1:2,000 dilution for 1 h at 25°C. Next, the specific protein bands were visualized using Chemiluminescence ECL kit (Millipore) and detected by GE Healthcare AI600 Imager (General Electric Company).

### Q-PCR Analysis

RNA was extracted using TRIZOL (Invitrogen). Total RNA concentration was quantified using the Multimode Microplate Reader (TECAN), following which cDNA was synthesized using the 1st strand synthesis system (Roche). The expression of target genes was detected using Power SYBR Green (Roche). The following primer sequences were used:

Human YBX3 Forward: ACCGGCGTCCCTACAATTAC;
Human YBX3 Reverse: GGTTCTCAGTTGGTGCTTCAC.
Human E-cadherin Forward: CGAGAGCTACACGTTCACGG;
Human E-cadherin Reverse: GGGTGTCGAGGGAAAAATAGG.
Human Vimentin Forward: AGTCCACTGAGTACCGGAGAC;
Human Vimentin Reverse: CATTTCACGCATCTGGCGTTC.
Human MMP1 Forward: AAATGCAGGAATTCTTTGGG;
Human MMP1 Reverse: ATGGTCCACATCTGCTCTTG.
Human GAPDH Forward: GGAGCGAGATCCCTCCAAAAT;
Human GAPDH Reverse: GGCTGTTGTCATACTTCTCATGG.

### Cell Wound Scratch Assay

The 5-8F and S18 YBX3 control or YBX3 shRNA cells were cultured in six-well plates. When the cells grew to 100% confluence, a scratch was made in each well using a 200 μl sterile pipette tip. After washing the scraped cells with PBS, the cells were cultured in fresh media for 48 h. Images were acquired using Discover Echo (Echo), and the gap distances were quantified using Image J.

### Cell Migration Assay

The 24 wells transwell chambers with 8.0 pore membranes (Corning USA) were applied according to the manufacturer’s protocol. RPMI 1640 or DMEM (600 μl) with 20% FBS were added in the lower compartment. The 5-8F and S18 YBX3 control or YBX3 shRNA cells were seeded in the upper compartment at a density of 5 × 10^4^ cells/100 μl/well. After incubated for 24 h, the cells in chambers were fixed with 4% PFA, stained with crystal violet (Macklin), and images were acquired using Discover Echo (Echo). The migrating cells were counted using Photoshop.

### Immunohistochemical (IHC) Analysis

The Paraffin-embedded tumor sections were deparaffinized in xylene (National Diagnostics) and rehydrated in a graded alcohol series permeabilized in PBS with 0.5% Triton-X (Sigma). H&E staining was performed following standard protocol. For staining with specific antibodies, the sections were boiled in sodium citrate for antigen retrieval and then after incubated with a blocking buffer (Vector). Primary antibodies against YBX3 (Immuno-Biological Laboratory) were added at 1:100 dilution and incubated at 4°C overnight. Next, the slides were washed by PBS, and incubated with HRP-labeled secondary antibody (Vector). DAB was used to develop the peroxidase reaction product. After washing, the sections were stained with hematoxylin, and the images were captured using an automatic scanning microscope Motic BA600-4 (Motic).

### RNA Sequencing Analysis

After transfecting 5-8F cells by lentivirus shRNA-YBX3 or shRNA-control. The total RNA of each group was extracted and sequenced on the Illumina HiSeq/MiSeq platform at Novogene Co. Ltd. RNA-seq results read in fasta format after fastp data quality evaluation and filtering. DESeq2 was used to analyze differentially expressed genes (DEGs) with cutoff values of padj_value <0.05 and a 2.0 fold change. The DEGs were subjected to KEGG pathway analysis using GO-seq and p < 0.05 as the cutoff value.

### Statistical Analysis

Graphpad Prism 7.0 was used for the statistical analysis. All values are represented as the mean ± SEM. The statistical significance was determined using the Student’s *t*-test or one-way ANOVA, followed by Dunnett’s multiple comparison test. Values were considered statistically significant at *p* < 0.05.

## Results

### High Expression of YBX3 in NPC Is Associated With Metastasis

**Figure 1:**
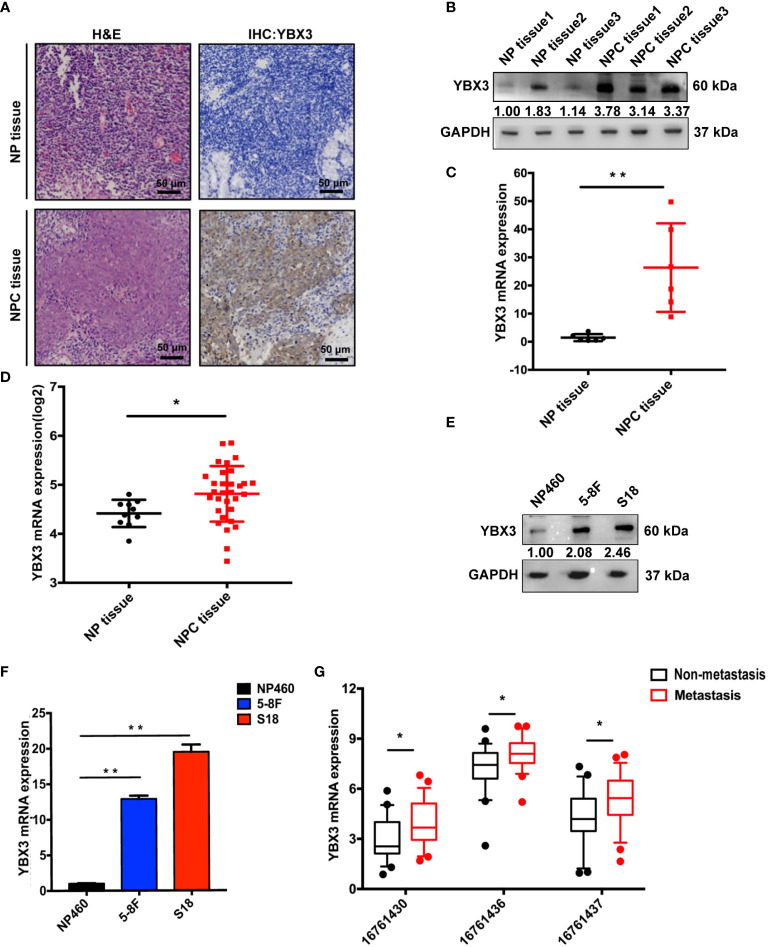
High expression of YBX3 in NPC tissues and cell lines. **(A)** Representative histologic sections of nasopharyngeal tissue and nasopharyngeal carcinoma tissue in patients were strained with H&E and IHC-YBX3. H&E demonstrated a typical feature of Nasopharyngeal Carcinoma, and the expression of YBX3 protein was detected in the tumor cells. Scale bar, 50 μm. **(B)** WB analysis was used to detect the protein level expression of YBX3 and in normal nasopharyngeal tissues and patient derived nasopharyngeal carcinoma tissue. **(C)** Q-PCR analysis was used to detect the RNA level expression of YBX3 in normal nasopharyngeal tissues and patient derived nasopharyngeal carcinoma tissue. **p < 0.01. **(D)** YBX3 mRNA expression level in NPC tissues and normal tissues from Rhodes’s dataset in ONCOMINE database (n = 31). *p < 0.05. **(E)** WB analysis was used to detect the protein level expression of YBX3 in NP460 and NPC cells with 5-8F and S18. **(F)** Q-PCR analysis was used to detect the RNA level expression of YBX3 in NP460 and NPC cells with 5-8F and S18. **p < 0.01. **(G)** YBX3 expression levels in NPC tumor tissues with and without distant metastasis from GSE103611 in GEO database (n = 24). Three probes: 16761430, 16761436, and 16761437 targeted different positions of YBX3. *p < 0.05. NP tissue, normal nasopharyngeal tissues; NPC tissue, nasopharyngeal carcinoma tissues.

We first compared the expression of YBX3 in NPC and normal nasopharyngeal tissues. IHC analysis revealed that the protein expression of YBX3 in NPC tissues was significantly higher than that in normal nasopharyngeal tissues (**Figure 1A**), due to the stronger reactivity of YBX3 antibody in tumor cells. WB and Q-PCR analysis of fresh-frozen clinical samples also showed that the protein and mRNA expressions of YBX3 were markedly upregulated in NPC tissues, compared to their expression in the normal nasopharyngeal tissues (**Figures 1B, C**). In addition, the high expression of YBX3 in NPC tissues from ONCOMINE database also supported the above results (**Figure 1D**). Consistent with the increase in clinical samples, the elevated expression of YBX3 was also observed in NPC cell lines 5-8F and S18, compared with that noted in the human normal nasopharyngeal epithelial cell NP460 (**Figures 1E, F**). These findings suggested that YBX3 expression levels were increased in NPC tissues and the 5-8F and S18 cells with the high malignancy and metastasis.

We next analyzed the microarray data of NPC tumor tissues with and without distant metastasis from GEO database to determine whether the expression level of YBX3 was related to NPC metastasis (25). As expected, the results of the three probes (16761430, 16761436, and 16761437) targeting different positions of YBX3 were all revealed that the expression of YBX3 was higher in NPC tissues with distant metastasis than those without metastasis (**Figure 1G**). These findings suggested that high expression of YBX3 in NPC was associated with metastasis. Thus, we hypothesized that YBX3 may be associated with the metastasis of NPC.

### Depletion of YBX3 Represses the Migration of NPC Cells *In Vitro*

**Figure 2:**
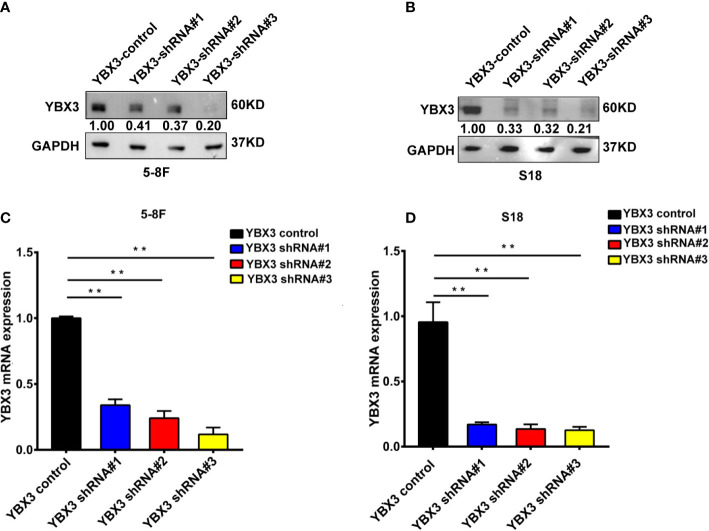
The efficiency of YBX3 in NPC cells interfered by lentiviruses. **(A)** WB analysis was used to detect the interfering effects of YBX3 shRNA in 5-8F. **(B)** Q-PCR analysis was used to detect the interfering effects of YBX3 shRNA in 5-8F. **(C)** WB analysis was used to detect the interfering effects of YBX3 shRNA in S18. **p < 0.01. **(D)** Q-PCR analysis was used to detect the interfering effects of YBX3 shRNA in S18. **p < 0.01.

Subsequently, we determined the effects of YBX3 on NPC cells migration following YBX3 knockdown with lentivirus shRNAs. As shown in **Figures 2A–D**, mRNA and protein expression levels of YBX3 were sharply decreased after transfected with three shRNAs targeting different positions of YBX3 (YBX3-shRNAs), respectively, among which YBX3 shRNA#3 has the most knockdown efficient on YBX3 expression. Therefore, YBX3 shRNA#3 was used for further functional and the mechanistic investigations.

**Figure 3:**
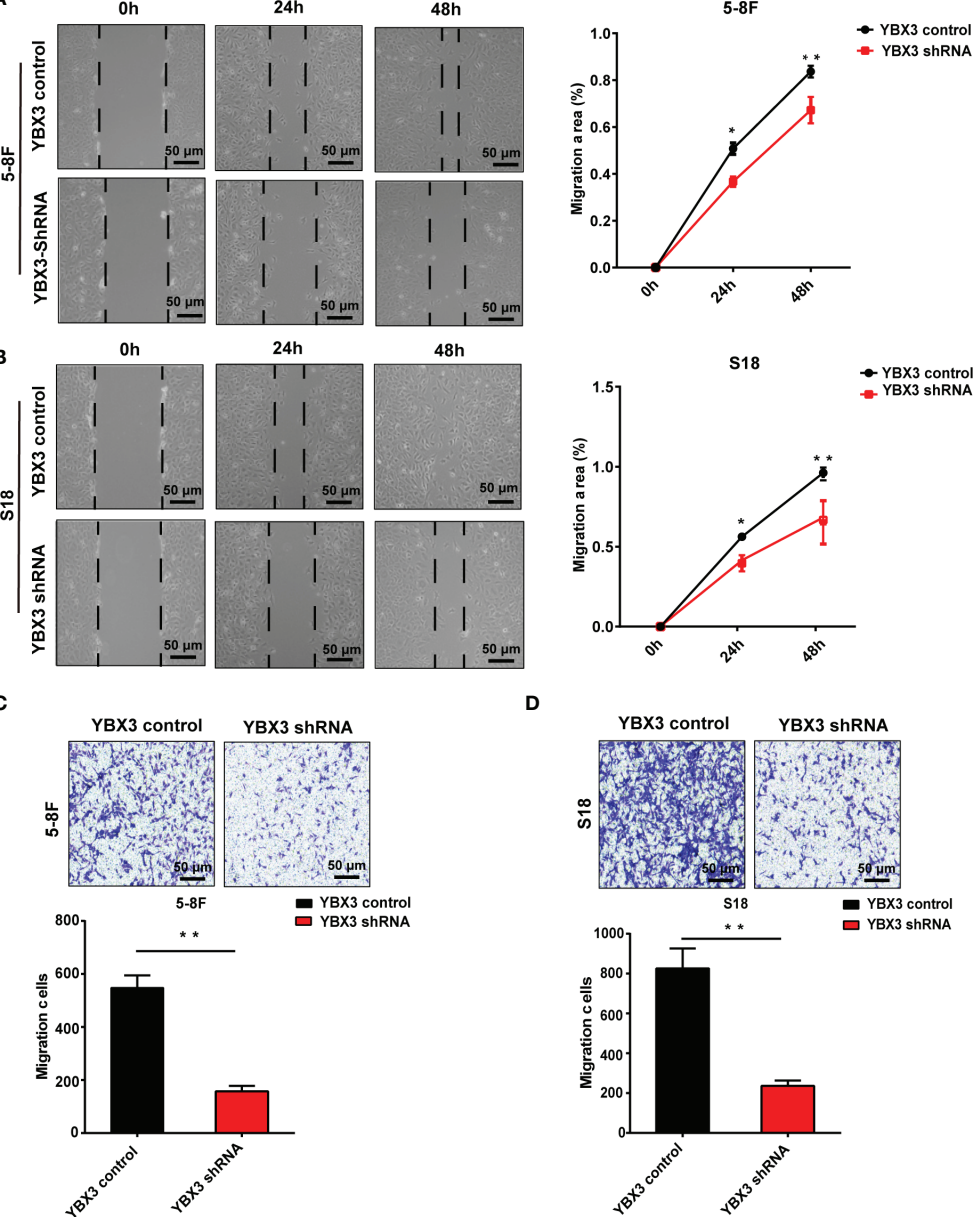
Deficiency of YBX3 inhibits NPC cell migration. **(A)** The wound healing experiments was used to detect the migration ability in 5-8F. Scale bar, 50 μm; *p < 0.05; **p < 0.01. **(B)** The wound healing experiments were used to detect the migration ability in S18. Scale bar, 50 μm; *p < 0.05; **p < 0.01. **(C)** The transwell experiments were used to detect the migration ability in 5-8F. Scale bar, 50 μm; **p < 0.01. **(D)** The transwell experiments were used to detect the migration ability in S18 cells. Scale bar, 50 μm; **p < 0.01.

Intriguingly, the results from scratch wound healing showed that the migration ability of 5-8F and S18 cells were both significantly reduced following 48 h in YBX3 shRNA transfected cells compared with YBX3 control transfected cells. And the inhibitory effects of YBX3 knockdown on cell migration were more pronounced the longer the cells were cultured (**Figures 3A, B**). Similarly, transwell migration assay demonstrated that YBX3 depletion also decreased the numbers of migration cells by 73 and 74% in 5-8F and S18 cells, respectively, compared with YBX3 control transfected cells (**Figures 3C, D**). Thus, our *in vitro* data indicated that YBX3 may be involved in cell migration in NPC.

### Depletion of YBX3 Regresses NPC Metastasis *In Vivo*

**Figure 4:**
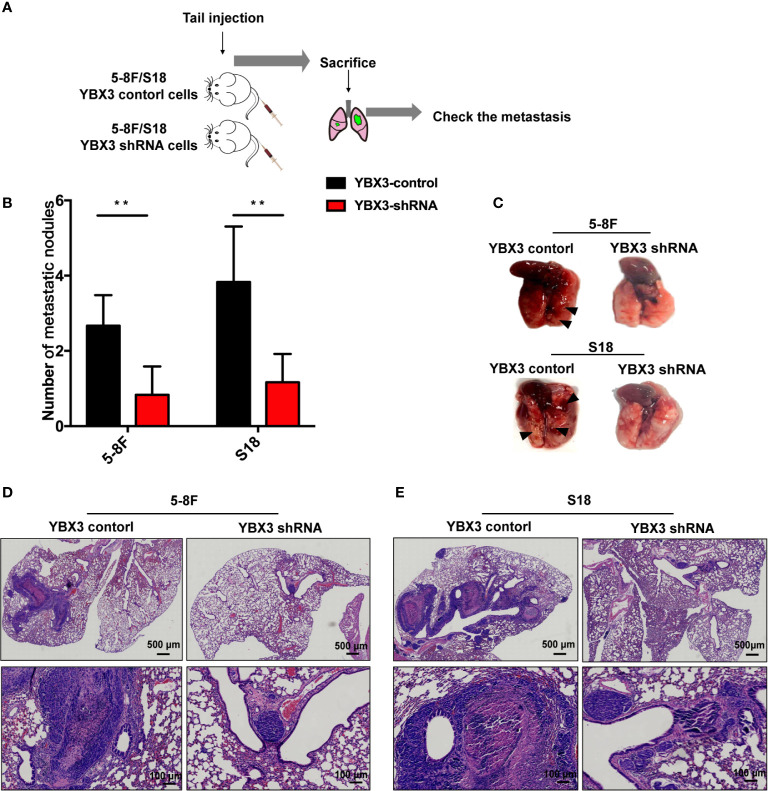
YBX3 affects NPC tumor metastasis. **(A)** Schematic diagram depicting the lung metastasis model of NPC by injecting 5-8F and S18 cells with stably depleting YBX3 into the tail vein. **(B)** The visible surface lung metastatic nodules were quantified with six lungs per group (n = 6). **p < 0.01. **(C)** Representative images of lung and quantifications of YBX3 control and YBX3 shRNA metastatic cancer models. Arrowheads point to metastatic nodules. **(D, E)** H&E staining analysis of lung metastasis. Scale bar, 500 μm (up); 100 μm (down).

To further validate the effects of YBX3 on NPC metastasis *in vivo*, we subcutaneously performed the lung metastatic mice model by injecting 5-8F and S18 cells with stably depleting YBX3 into the tail vein of nude mice (**Figure 4A**). Thirty days later, the mice were sacrificed, and the metastatic tumor nodules in the lung were evaluated. As expected, the mice injected with YBX3-depleting cells had fewer lung metastasis nodules than the mice injected with control cells (**Figures 4B, C**). Meanwhile, lung metastatic nodule in the mice injected with YBX3-depleting cells exhibited a smaller area than those injected with the control cells (**Figures 4D, E**). Therefore, our *in vivo* results indicated that YBX3 plays an important role in metastasis of NPC cells.

### YBX3 Involves in NPC Metastasis *via* PI3K/AKT Signaling

**Figure 5:**
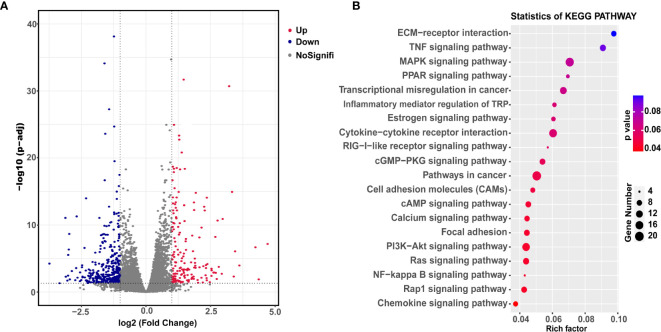
Downstream transcriptional genes of YBX3 in NPC cells. **(A)** Volcano diagram depicting the 558 differentially expressed genes (DEGs) from the RNA sequencing in 5-8F cells transfected with shRNA-YBX3 or shRNA-control. Red represents upregulated genes, blue represents downregulated genes, gray represents no-signification. **(B)** KEGG pathway analysis of the DEGs between shRNA-YBX3 and shRNA-control groups.

Finally, we explored the underlying mechanism of YBX3 in NPC metastasis. RNA sequencing analysis was conducted in 5-8F cells transfected with shRNA-YBX3 or shRNA-control. As shown in **Figure 5A**, a total of 558 DEGs were identified (379 downregulated genes and 179 upregulated genes) between shRNA-YBX3 and the negative control groups. Moreover, the DEGs were subjected for KEGG pathway analysis. We found that the DEGs were significantly enriched in the following pathways: cell adhesion molecules, cAMP signaling pathway, calcium signaling pathway, focal adhesion, PI3K/AKT signaling pathway, Ras signaling pathway, Rap1 signaling pathway, NF-κB signaling pathway, and Chemokine signaling pathway (**Figure 5B**). Of these, PI3K/AKT signaling pathway contained the most genes (**Figure 5B**).

**Figure 6:**
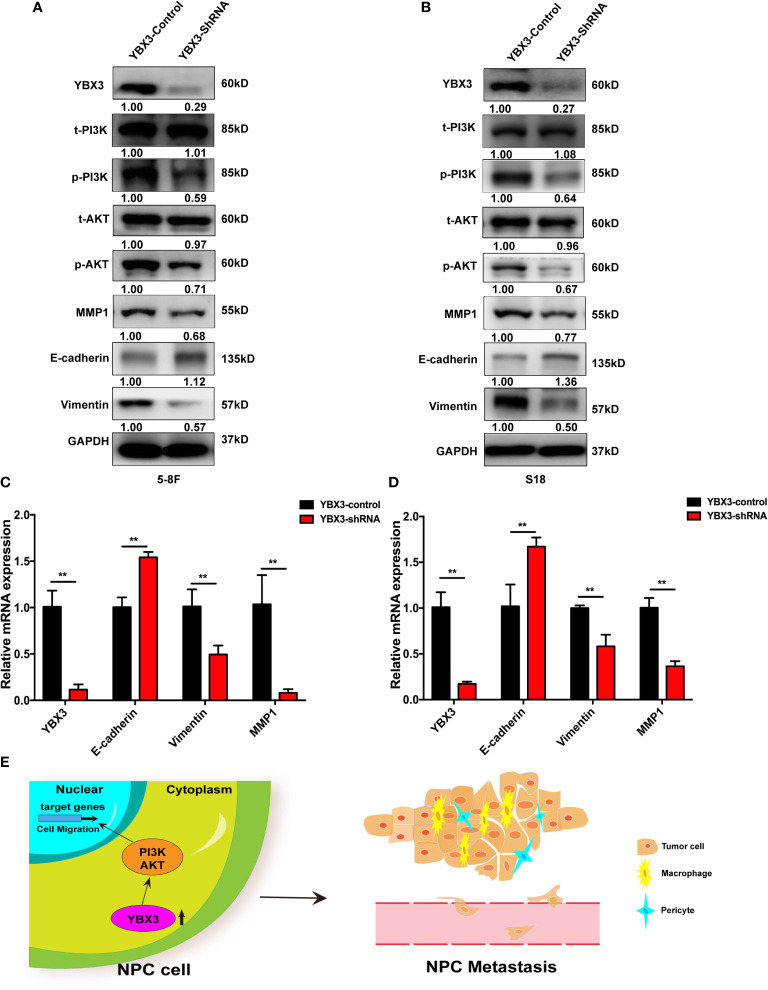
YBX3 promotes NPC metastasis by activating PI3K/AKT signaling. **(A)** WB analysis was used to detect the activation of PI3K/AKT signaling and cell migration related marker molecules in 5-8F. **(B)** WB analysis was used to detect the activation of PI3K/AKT signaling and cell migration related marker molecules in S18. **(C)** Q-PCR analysis was used to detect the activation of PI3K/AKT signaling and cell migration related marker molecules in 5-8F. **(D)** Q-PCR analysis was used to detect the activation of PI3K/AKT signaling and cell migration related marker molecules in S18. **p < 0.01. **(E)** Schematic diagram depicting the YBX3 mediated regulation of target genes *via* PI3K/AKT signaling, which is involved in the modulation of NPC metastasis.

Since the activation of PI3K/AKT signaling has been reported to play a crucial role in cell migration of NPC (26, 27). Subsequently, to verify the pathway analysis results, the levels of total-PI3K(t-PI3K), total-AKT (t-AKT) and phosphorylated-PI3K (p-PI3K), phosphorylated-AKT (p-AKT) were monitored with western blot after depleting YBX3 with shRNA in 5-8F. As shown in **Figure 6A**, obvious decrease of p-PI3K and p-AKT levels occurs due to the knockdown of YBX3 in 5-8F cells compared to the control cells, although no significant change in t-PI3K and t-AKT levels were observed. This suggested that YBX3 knockdown suppressed the activity of PI3K/AKT signaling in 5-8F cells. Accordingly, the protein and mRNA levels of E-cadherin were increased and the expression of MMP1 and Vimentin were decreased after depleting YBX3 in 5-8F cells (**Figures 6A–C**). Similarly, YBX3 knockdown inhibited the PI3K/AKT signaling activity and changed the expression levels of migration-related molecules, as confirmed in S18 cells (**Figures 6B, D**). Therefore, the above data manifest that YBX3 functions in NPC metastasis at least partly through the regulation of PI3K/AKT signaling pathway (**Figure 6E**).

## Discussion

YBX3 has been reported to play an important role in many functions of cell and various diseases, which may be harnessed for digging into the mechanisms of NPC metastasis. For example, YBX3 was upregulated in SW620 cells and promoted the development of CRC through activating TAK1, p38, and JNK (28). In skin squamous cell carcinoma (SCC), YBX3 was high expression in SCC patient tissues and cells, and promoted the proliferation, migration, and invasion of SCC cells depending on the NF-κB pathway (29). However, the expression and biological functions of YBX3 in NPC, especially in NPC metastasis have not yet been fully understood. Here, we firstly demonstrated that YBX3 was highly expressed in NPC tissues and cell lines, and associated with NPC metastasis, suggesting that YBX3 may function in the development of NPC. To confirm this, YBX3 expression was knocked down by lentivirus shRNA and subsequently founding proved that YBX3 expression depleting decreased NPC cell migration *in vitro* and metastasis *in vivo*.

In addition, growing evidences indicated PI3K/AKT pathway is associated with cell growth, survival, differentiation, glucose transport and metabolism, which activate and phosphorylate downstream proteins to perform biological functions (30–32). Moreover, the PI3K/AKT pathway has been widely demonstrated to play pivotal role in tumor progression and metastasis in various cancers, such as NPC (33), thyroid cancer (34), hepatocellular carcinoma (35), pancreatic cancer (36), and so on. In chronic myeloid leukemia, the YBX3 was shown to be a regulator of proliferation in the CML cell line through the PI3K/AKT/Bcr/Abl pathway (37). YBX3 and YBX1could convey profound anti- and pro-oncogenic effects on AKT pathway dependent cell transformation (38). In gynecological cancer, YBX1 was shown to be an important player impacting AKT and the downstream genes such as EGFR, Snail, or E-cadherin (39). Thus, these findings suggested that YBX3 and YBX superfamily were closely associated with PI3K/AKT pathway.

Mechanistically, PI3K/AKT pathway is reported to decrease the expression of E-cadherin and to increase the expression of N-cadherin, thereby regulating the EMT phenotype, results in promoting cell migration (40, 41). Meanwhile, matrix metalloproteinases also play an essential role in many tumors, high expression of MMP1 were closely associated with tumor metastasis (42). Here we found that deficiency of the YBX3 could remarkably inhibit the activation of PI3K/AKT signaling, suppress EMT and MMP1 by increasing E-cadherin expression and decreasing Vimentin and MMP1 level in NPC cell lines. Hence, YBX3 functions in NPC metastasis at least partly through the regulation of PI3K/AKT signaling pathway.

However, several points are worth noting in our study. The number of clinical tissue samples need to expand and the clinical metastatic NPC tissue samples need to be studied. Additionally, although the molecular mechanism indicated that YBX3 can regulate the expression of cell migration related markers, the detailed underlying regulatory mechanisms require to be further explored.

In conclusion, our findings demonstrated that YBX3 plays an important role in the metastasis of NPC. Deficiency of YBX3 expression in NPC cells reduced tumor metastasis by activating PI3K/AKT signaling *in vitro* and *in vivo*. These results suggest that YBX3 may be a novel biomarker for tumor metastasis and a potential biomarker for treatment of NPC.

## Data Availability Statement

The datasets used and/or analyzed during the present study are available from the ONCOMINE gene expression array datasets (www.oncomine.org), and the GEO database (Accession number: GSE103611 and GSE160245).

## Ethics Statement

The studies involving human participants were reviewed and approved by the Ethics Committee of the Institutional Review Board of Shenzhen Second People’s Hospital. The patients/participants provided their written informed consent to participate in this study. The animal study was reviewed and approved by the Animal Ethics Committee of Shenzhen University.

## Author Contributions

GN and XF designed the study. XX, YW, HW, and TD performed the experiment. MY collected the clinical patient sample. XW performed the pathological experiment and analysis. WW proofread the manuscript. All authors contributed to the article and approved the submitted version.

## Funding

This work was supported by the National Natural Science Foundation of China (grant number: 81902777), Natural Science Foundation of Guangdong Province under Grant (grant number: 2018A0303130295), and Shenzhen Science and Technology Innovation Committee under Grant (grant number: JCYJ20170302165727389).

## Conflict of Interest

The authors declare that the research was conducted in the absence of any commercial or financial relationships that could be construed as a potential conflict of interest.
